# Molecular weaponry: diverse effectors delivered by the Type VI secretion system

**DOI:** 10.1111/cmi.12532

**Published:** 2015-11-03

**Authors:** Juliana Alcoforado Diniz, Yi‐Chia Liu, Sarah J. Coulthurst

**Affiliations:** ^1^ Division of Molecular Microbiology, School of Life SciencesUniversity of DundeeDundeeDD1 5EHUK

## Abstract

The Type VI secretion system is a widespread bacterial nanomachine, used to deliver toxins directly into eukaryotic or prokaryotic target cells. These secreted toxins, or effectors, act on diverse cellular targets, and their action provides the attacking bacterial cell with a significant fitness advantage, either against rival bacteria or eukaryotic host organisms. In this review, we discuss the delivery of diverse effectors by the Type VI secretion system, the modes of action of the so‐called ‘anti‐bacterial’ and ‘anti‐eukaryotic’ effectors, the mechanism of self‐resistance against anti‐bacterial effectors and the evolutionary implications of horizontal transfer of Type VI secretion system‐associated toxins. Whilst it is likely that many more effectors remain to be identified, it is already clear that toxins delivered by this secretion system represent efficient weapons against both bacteria and eukaryotes.

## Introduction

**Figure 1:**
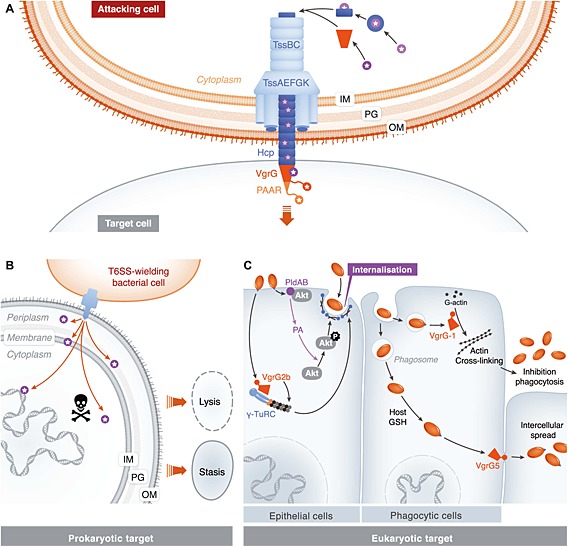
The Type VI secretion system and its action against prokaryotic and eukaryotic target cells. A. Schematic depiction of the T6SS firing into a target cell, with components of the expelled puncturing device highlighted in colour (green, Hcp; red, VgrG; orange, PAAR), and effectors or effector domains shown with stars. Cargo effectors are recruited by interaction with Hcp or VgrG, whereas specialized effectors contain effector domains fused to VgrG or PAAR. B. General representation of an anti‐bacterial T6SS, where multiple toxic effectors attack targets in the periplasm, membrane and/or cytoplasm of a rival bacterial cell, leading to inhibition of growth or lysis. C. Schematic illustrating how the best characterized T6SS‐delivered anti‐eukaryotic effectors interfere with eukaryotic host biological functions. In epithelial cells, *P*. *aeruginosa* promotes bacterial internalization by delivering phospholipases PldA and PldB, which induce the activation of the PI3K/Akt pathway, and VgrG2b, which binds to *γ*‐tubulin ring complex (*γ*TuRC). The Pld proteins bind to Akt and also their enzymatic activity releases phosphatidic acid (PA), which is likely the signal inducing Akt phosphorylation. In phagocytic cells, *V. cholerae* delivers VgrG‐1 from the phagosome to the cytosol where it inhibits further phagocytosis by cross‐linking actin, whereas *Burkholderia* utilize another specialized VgrG, VgrG5, to induce host cell membrane fusion, promoting mononuclear giant cell formation and intercellular spread. OM, outer membrane; PG, peptidoglycan; IM, inner membrane.

Bacteria utilize protein secretion systems to deliver specific proteins to the extracellular environment or directly into target cells. Protein secretion systems, and the proteins they translocate, play key roles in the interactions of bacterial cells with host organisms, competitor bacteria and the abiotic environment. The Type VI secretion system (T6SS) is widespread in Gram‐negative bacteria and targets both eukaryotic and prokaryotic cells, in a contact‐dependent manner. The T6SS nanomachine is composed of 13 conserved components forming the core machinery (TssA–M), together with variable accessory proteins. Our current model of the structure and mode of action of the T6SS, which exhibits striking parallels with the cell puncturing machinery of contractile bacteriophage tails, has been reviewed recently (Ho *et al.*, 2014; Zoued *et al.*, 2014). In brief, contraction of a tail sheath‐like structure made up of TssBC propels (‘fires’) a puncturing structure made of Hcp (TssD) and VgrG (TssI) out of the secreting cell and into a target cell. The expelled puncturing structure consists of a tube of stacked Hcp hexamers, topped with a VgrG trimer ‘spike’ and a final sharp ‘tip’ from a PAAR protein (Fig. 1A). The cytoplasmic sheath is anchored in a membrane‐associated basal complex made up of a cytoplasmic baseplate (TssAEFGK) and a membrane complex (TssJLM). Following ‘firing’, the sheath is disassembled by the cytoplasmic ATPase TssH (ClpV), allowing recycling of the machinery for further rounds of secretion. As discussed in the succeeding paragraphs, secreted proteins are translocated into target cells by association with components of the expelled Hcp‐VgrG‐PAAR structure.

Varied proteins delivered by the T6SS, known as ‘effectors’, have now been identified and shown to present various toxic activities in target cells. Clearly, characterising the repertoire of effectors delivered is central to understanding the biological role of the T6SS. Indeed the importance of the T6SS and its distinctive roles in different bacterial interactions (Fig. 1B and C) are becoming increasingly appreciated. In some cases, T6SSs that target eukaryotic cells are required for virulence in the host organism, for example, in *Vibrio cholerae* and *Burkholderia* species (Ma and Mekalanos, 2010; Burtnick *et al.*, 2011; Schwarz *et al.*, 2014), or can mediate interactions with eukaryotic cells (Weyrich *et al.*, 2012; Jiang *et al.*, 2014). In many other cases, bacteria with so‐called ‘anti‐bacterial’ T6SSs should gain a fitness advantage in mixed microbial communities, as a consequence of the injection of anti‐bacterial toxins into rival species (Hood *et al.*, 2010; MacIntyre *et al.*, 2010; Murdoch *et al.*, 2011). Anti‐bacterial T6SSs can also confer intra‐species competitiveness (Unterweger *et al.*, 2014; Alcoforado Diniz and Coulthurst, 2015), competitive persistence in mixed‐species biofilms (Schwarz *et al.*, 2010) and even co‐operative, self‐recognition behaviour (Wenren *et al.*, 2013). Cells elaborating an anti‐bacterial T6SS adopt a self‐protection system utilising specific immunity proteins to neutralise the cognate toxins and prevent self‐intoxication or sibling‐intoxication.

In this article, we review recent advances in understanding T6SS‐delivered effector proteins, considering both their modes of delivery and their modes of action against prokaryotic cells, eukaryotic cells or even both. We also briefly consider the evolution of T6SS‐dependent toxins and emphasize the contribution of their deployment to fitness advantage in diverse microbial communities and to successful establishment of infection in the host.

## Multiple routes of effector delivery

Recent work has begun to shed light on how effectors are recruited and delivered by the T6SS. The current model of T6SS (Fig. 1A) allows classification of effectors according to their broad mode of delivery, into ‘cargo’ or ‘specialized’ effectors. Cargo effectors interact with components of the expelled puncturing structure, namely, Hcp, VgrG or PAAR, for delivery. In contrast, specialized effectors consist of effector domains covalently fused to one of these components, with the specialized effector typically present as one of several homologues of that core component (Durand *et al.*, 2014).

Hcp appears to have a multi‐faceted role in the T6SS. Silverman and co‐workers first showed that certain effectors can interact with the interior of the hexameric Hcp ring, allowing Hcp to direct their secretion. Furthermore, Hcp can define substrate specificity and promote the intracellular stability of these cargo effectors (Silverman *et al.*, 2013; Whitney *et al.*, 2014). It appears that Hcp proteins might also be capable of functioning as specialized effectors, because a gene encoding an Hcp‐like protein fused with an additional putative effector domain has been identified in *Salmonella enterica* Arizonae (Blondel *et al.*, 2009). VgrG proteins are another class of structural components able to interact with specific cargo effectors, as shown for VgrG‐1 and TseL in *V. cholerae* (Liang *et al.*, 2015; Unterweger *et al*., 2015), and supported by genomic evidence that *vgrG* genes are often found adjacent to putative effector genes (Barret *et al.*, 2011; Russell *et al.*, 2013). Alternatively, many VgrG‐based specialized effectors, originally termed ‘evolved VgrGs’, have now been described, possessing a variety of effector domains at their C‐termini (Pukatzki *et al.*, 2007; Brooks *et al.*, 2013). The final class of core components that can be involved in specific effector delivery are the recently discovered PAAR proteins (Shneider *et al.*, 2013). These proteins could carry cargo effectors, although this has not yet been shown. However, they appear to frequently act as specialized effectors, when PAAR domains are present within larger proteins, including Rhs proteins (Hachani *et al.*, 2014; Whitney *et al.*, 2014). T6SS‐associated Rhs proteins contain PAAR domains in their N‐terminal region, responsible for binding to VgrG, long Rhs repeat regions and variable C‐terminal toxin (effector) domains (Koskiniemi *et al.*, 2013; Alcoforado Diniz and Coulthurst, 2015).

Nevertheless, it is becoming clear that delivery of effectors can be more complex than the simple model just described. A new class of accessory protein, which mediates the interaction between VgrG proteins and effectors in certain T6SSs, has been reported recently: the ‘T6SS effector chaperones’ or ‘Type VI adaptor proteins’, containing DUF4123 domains. These proteins are not required for T6SS activity, but appear to interact with their cognate effector protein and a specific VgrG to allow delivery of the effector, exemplified by VC1417 assisting VgrG‐1 dependent secretion of TseL in *V. cholerae* (Liang *et al.*, 2015; Unterweger *et al*., 2015). Another member of this family, VasW, was earlier described as playing a crucial role in the secretion of the VasX effector (Miyata *et al.*, 2013). Alternatively, a domain found in the N‐terminal region of a subset of T6SS effectors, named MIX (marker for Type VI effectors), is proposed to represent a binding region for VgrG (Salomon *et al.*, 2014). Another, unrelated, family of conserved accessory proteins termed EagR (effector‐associated gene, with Rhs), formerly assigned DUF1795, has also been reported. EagR proteins are very often encoded upstream of *rhs* genes and EagR1 is specifically required for Rhs1‐dependent bacterial killing in *Serratia marcescens* (Alcoforado Diniz and Coulthurst, 2015). Overall, the existence of a number of basic modes of effector recruitment, further refined using more specific accessory proteins, likely explains how the T6SS is able to deliver numerous and structurally diverse proteins.

## Diverse and potent anti‐bacterial toxins delivered by the Type VI secretion system

**Figure 2:**
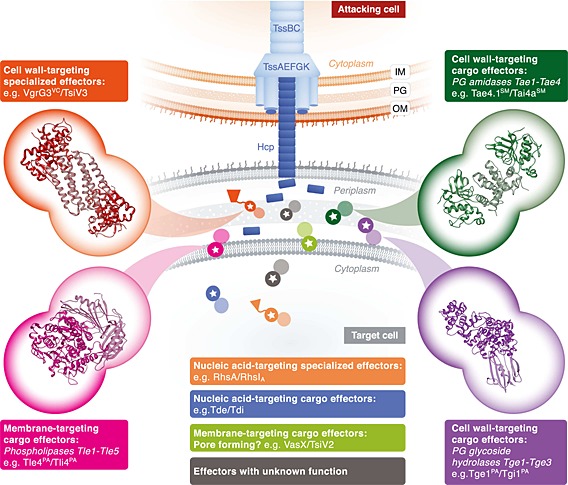
Anti‐bacterial Type VI secreted effectors: cellular targets and self‐protection mechanism. Schematic depiction of different classes of effector toxins and their sites of action once delivered into a target cell. Because this is a resistant (sibling) cell, specific immunity proteins bind to their cognate effectors in order to neutralize their activity. Dark circles with stars represent effectors, whilst lighter circles represent corresponding immunity proteins. Examples of atomic structures of effector : immunity complexes for four classes of effector are shown in the insets (PDB entries: Tle4^PA^/Tli4^PA^, 4R1D; VgrG3^VC^/TsiV3, 4NOO; Tge1^PA^/Tgi1^PA^, 4N88 and Tae4.1^SM^/Tai4a^SM^, 4BI8; Table 1). Black circles represent anti‐bacterial effectors whose function is currently unknown. OM, outer membrane; PG, peptidoglycan cell wall; IM, inner membrane.

It is now clear that the T6SS can deliver multiple anti‐bacterial toxins with different sites of action in a target cell (Fig. 2). The first effectors with toxic activity against other bacteria were identified in *Pseudomonas aeruginosa* and named Tse1–3 (Hood *et al.*, 2010). Subsequent work revealed that Tse1 and Tse3 have bacteriolytic activity, destroying the cell wall via peptidoglycan amidase or muramidase activity, respectively (Russell *et al.*, 2011). Indeed the peptidoglycan cell wall now appears to be a common target of T6SS effectors. Russell and co‐workers described four phylogenetically distinct families of T6SS‐delivered amidase effectors (Tae), Tae1–Tae4, starting from a bioinformatic approach (Russell *et al.*, 2012). Structural and biochemical studies of the Tse1 effector, which belongs to the Tae1 family, revealed that it displays unrestricted access to the active site, consistent with a broad‐spectrum toxin rather than housekeeping function (Chou *et al.*, 2012; Ding *et al.*, 2012). However, the diversity within the Tae effectors may be even greater, since two Tae4 family effectors delivered by the T6SS of *S. marcescens*, Ssp1 (Tae4.1^SM^) and Ssp2 (Tae4.2^SM^), display non‐redundant substrate specificity and phenotypic consequences (English *et al.*, 2012; Srikannathasan *et al.*, 2013). The cell wall‐targeting effectors additionally include three families of peptidoglycan glycoside hydrolase (muramidase) effectors, Tge1–3. Tge1 is exemplified by Tse3 of *P. aeruginosa* whilst Tge2 of *Pseudomonas protegens* has also been shown to be a T6SS‐delivered anti‐bacterial effector (Russell *et al.*, 2011; Whitney *et al.*, 2013). Whilst the aforementioned are all cargo effectors, the C‐terminal domain of the specialized VgrG‐3 of *V. cholerae* is also a peptidoglycan hydrolase (Brooks *et al.*, 2013).

**Table 1:** Type VI secretion system‐associated immunity proteins with reported structures, either in complex with the cognate effector or alone.

Effector/immunity	Alternate name	Effector activity	Complex structure	Complex stoichio‐metry^a^	Immunity only^b^	Organism
Tae1^PA^/Tai1^PA^	Tse1/Tsi1	Peptidoglycan amidase	PDB 3VPJ (Ding *et al.*, 2012) [PDB 4FGI, 4EQA]^c^	1E:1I		*Pseudomonas aeruginosa*
Tae3^RP^/Tai3 ^RP^		Peptidoglycan amidase	PDB 4HZB (Dong *et al.*, 2013a)	2E:4I		*Ralstonia pickettii*
Tae4^ST^/Tai4^ST^		Peptidoglycan amidase	PDB 4HFF (Zhang *et al.*, 2013) [PDB 4J32]	2E:2I		*Salmonella typhimurium*
Tae4^EC^/Tai4^EC^		Peptidoglycan amidase	PDB 4HFL (Zhang *et al.*, 2013)	2E:2I		*Enterobacter cloacae*
Tae4.1^SM^/Tai4a^SM^	Ssp1/Rap1a	Peptidoglycan amidase	PDB 4BI8 (Srikannathasan *et al.*, 2013)	2E:2I		*Serratia marcescens*
Tae4.2^SM^/Tai4.2^SM^	Ssp2/Rap2a	Peptidoglycan amidase			PDB 3ZIB (Srikannathasan *et al.*, 2013)	*Serratia marcescens*
Rap1b, Rap2b		d			PDB 4AX2, 4B6I (English *et al.*, 2012)	*Serratia marcescens*
Tse2/Tsi2		Unknown			PDB 3RQ9 (Li *et al.*, 2012)	*Pseudomonas aeruginosa*
Tge1^PA^/Tgi1^PA^	Tse3/Tsi3	Peptidoglycan glycoside hydrolase	PDB 4N88 (Lu *et al.*, 2014a) [PDB 4LUQ, 3WA5]	1E:1I		*Pseudomonas aeruginosa*
Tge2^PP^/Tgi2^PP^		Peptidoglycan glycoside hydrolase	PDB 4KT3 (Whitney *et al.*, 2013)	1E:1I		*Pseudomonas protegens*
VgrG3^VC^/TsiV3	VgrG‐3/TsaB	Peptidoglycan hydrolysis	PDB 4NOO (Yang *et al.*, 2014) [PDB 4NSR]	2E:2I		*Vibrio cholerae*
Tle4^PA^/Tli4^PA^		Phospholipase?	PDB 4R1D (Lu *et al.*, 2014b)	1E:1I		*Pseudomonas aeruginosa*

a E:I, indicates effector: immunity stoichiometry in the complexes.

b Structures of immunity proteins where there is no effector or complex structure available.

c PDB identifiers for independent structures of the same proteins are given in square brackets.

d Rap1b and Rap2b are ‘orphan’ immunity proteins of the Tai4 class.

A group of nucleic acid‐targeting cargo effectors has been described, based on work in *Agrobacterium tumefaciens*. These Tde proteins display DNase activity, dependent on a conserved HxxD motif (Ma *et al.*, 2014). Tse2 from *P. aeruginosa* is a cytoplasmic‐acting effector that acts as a potent inhibitor of target cell proliferation and is proposed to act as a ribonuclease (Li *et al.*, 2012). As mentioned earlier, Rhs proteins are polymorphic toxins and include members that are specialized T6SS effectors. Several such Rhs effectors have been shown to have a C‐terminal toxin domain with an HNH endonuclease motif and DNase activity (Koskiniemi *et al.*, 2013; Alcoforado Diniz and Coulthurst, 2015). The activities of the C‐terminal toxin domains of several other reported T6SS‐delivered Rhs effectors are yet to be determined. Interestingly, the toxic domain of Rhs effectors is likely sequestered prior to secretion by a shell‐like structure formed by the Rhs repeats, similar to BC component of insecticidal ABC toxins (Busby *et al.*, 2013).

Type VI secretion system‐delivered effectors can also target the bacterial membrane. A diverse superfamily of bacterial lipase/phospholipase effectors (Tle) has been reported, sub‐divided into Tle1–Tle5. The Tle1–Tle4 families exhibit the GXSXG motif, which is common in lipases, whilst Tle5 present a dual HXKXXXXD motif associated with phospholipase D enzymes (Russell *et al.*, 2013). Representatives of Tle1 and Tle2 were shown to have phospholipase A activity and to be T6SS‐dependent anti‐bacterial effectors, whilst Tle5‐type effectors from *P. aeruginosa* (PldA and PldB) are phospholipase D enzymes, which act as effectors against both bacterial and eukaryotic cells (Dong *et al.*, 2013b; Russell *et al.*, 2013; Jiang *et al.*, 2014). Moreover, effectors predicted to have pore‐forming activity, such as VasX in *V. cholerae* and PA14_69520 in *P. aeruginosa*, which both display similarity with pore‐forming colicins, are also important players in anti‐bacterial warfare (Miyata *et al.*, 2013; Hachani *et al.*, 2014).

Notably, there are many other T6SS‐dependent effectors, either experimentally identified or predicted based on genomic context, whose function is not known or readily predictable (Barret *et al.*, 2011; Russell *et al.*, 2012; Fritsch *et al.*, 2013; Salomon *et al.*, 2014; Unterweger *et al.*, 2014; Whitney *et al.*, 2014). This implies significant diversity of effectors and the promise of novel cellular targets yet to be identified.

## Actions of anti‐eukaryotic toxins delivered by the Type VI secretion system

Whilst T6SS‐dependent virulence‐related phenotypes have been quite widely reported, only a few anti‐eukaryotic effector proteins have been identified. To date, the well‐characterized T6SS effector proteins targeting eukaryotic cells are several specialized VgrG proteins and cargo phospholipases (Fig. 1C). The C‐terminal domain of VgrG‐1 of *V. cholerae* shares homology with the actin cross‐linking domain (ACD) of RtxA family toxins and catalyses the covalent cross‐linking of G‐actin (monomeric actin) in an ATP‐dependent manner (Pukatzki *et al.*, 2007). Following internalization of *V. cholerae* cells, VgrG‐1‐dependent actin cross‐linking prevents host cell cytoskeleton rearrangements, inhibiting phagocytosis and protecting extracellular bacteria from subsequent uptake by macrophages (Ma *et al.*, 2009). *In vitro* injection of purified VgrG1‐ACD into human fibroblasts induces actin aggregation (Pukatzki *et al.*, 2007), whilst *in vivo* actin‐cross linking caused by VgrG1‐ACD results in induction of inflammatory diarrhoea that facilitates the survival of *V. cholerae* in the murine intestine (Ma and Mekalanos, 2010). The C‐terminal domain of VgrG1 from *Aeromonas hydrophila* also modifies host actin, presenting an ADP ribosyltransferase activity that induces apoptosis via the activation of caspase 9 in HeLa cells (Suarez *et al.*, 2010a). VgrG2b of *P. aeruginosa* promotes bacterial internalization by non‐phagocytic cells through specific binding to the *γ*‐tubulin ring complex (Sana *et al.*, 2015). VgrG5 of *Burkholderia pseudomallei* and *Burkholderia thailandensis* is required for host cell fusion, and thus intercellular spread of bacteria. It is proposed that the C‐terminal domain may insert into host cell membranes to mediate the fusion event (Schwarz *et al.*, 2014; Toesca *et al.*, 2014).

Considering cargo effectors, recent studies have shown that T6SS‐dependent phospholipases can target eukaryotic as well as prokaryotic cells, revealing the versatility of such effectors across kingdoms. PldA of *P. aeruginosa* degrades phosphatidylethanolamine and was first identified as an H2‐T6SS‐dependent Tle5 family effector (Russell *et al.*, 2013). A subsequent study demonstrated that both PldA and another phospholipase D protein, PldB, which is delivered by H3‐T6SS*,* are able to promote internalization in human epithelial cells via the induction of the phophatidylinositol 3‐kinase (PI3K)/Akt pathway. PldB also acts as an anti‐bacterial toxin (Jiang *et al.*, 2014). Similarly, TseL (Tse2^VC^) of *V. cholerae* exerts both anti‐bacterial and anti‐eukaryotic activity (Dong *et al.*, 2013b). Additionally, phospholipase Pld1 in *Klebsiella pneumoniae*, encoded within a T6SS gene cluster and thus likely a T6SS effector, was required for virulence in a mouse pneumonia model (Lery *et al.*, 2014).

VasX of *V. cholerae*, another trans‐kingdom cargo effector protein, disrupts the inner membrane of bacterial cells and is also active against *Dictyostelium discoideum* (Miyata *et al.*, 2011; Miyata *et al.*, 2013). EvpP, secreted by the T6SS of *Edwardsiella tarda*, is required for virulence and proliferation in fish hosts although its mode of action is not yet known (Zheng and Leung, 2007; Wang *et al.*, 2009). Whilst non‐specialized Hcp is not an effector, rather a component of the secretion machinery, it is interesting to note several studies reporting immunogenic effects of this secreted protein. Recombinant Hcp from *A. hydrophila* is reported to bind to murine macrophage‐like cells RAW 264.7 and induce or inhibit the production of anti‐inflammatory or pro‐inflammatory cytokines respectively (Suarez *et al.*, 2010b). Similarly, one of two Hcp proteins associated with a T6SS in meningitis‐causing *Escherichia coli* is reported to induce cytoskeleton rearrangement, apoptosis and cytokine release in brain endothelial cells (Zhou *et al.*, 2012).

In general, T6SSs acting against eukaryotic cells appear to be appropriately regulated to promote host infections. For example, expression of the H2‐T6SS of *P.* aeruginosa is modulated by quorum sensing and induced by iron limitation (Sana *et al.*, 2012), and the activation of T6SS‐5, which delivers VgrG5, in *B. pseudomallei*, is triggered by the histidine kinase VirA upon sensing the host cytosolic glutathione, thereby facilitating bacterial spreading at the right time, namely, when the bacteria have escaped from the phagosome to the cytosol (Fig. 1C) (Wong *et al.*, 2015). Overall, recent work suggests widespread roles for anti‐eukaryotic T6SSs in host cell interactions and virulence, and that a multitude of T6SS dependent effectors acting against eukaryotic cells remain to be identified.

## Self‐protection against anti‐bacterial toxins: specific immunity proteins

All T6SS‐dependent anti‐bacterial effectors identified to date, both cargo and specialized, are encoded adjacent to a specific immunity protein. Immunity proteins reside in the compartment of action of the toxin (Fig. 2) and protect the secreting cell from effectors injected by neighbouring sibling cells or, in the case of cytoplasmic‐acting effectors, from its own effectors prior to secretion. Loss of an immunity protein renders a bacterium susceptible to T6SS‐mediated killing or inhibition by the cognate effector. This has provided a useful genetic means to identify T6 effectors, for example, in *V. cholerae* and *S. marcescens* (English *et al.*, 2012; Dong *et al.*, 2013b).

In all cases where the mechanism of self‐protection has been examined, there is a direct interaction between the effector and its cognate immunity protein. In other words, the immunity protein neutralizes the effector rather than protecting the target. A more detailed picture of how effector toxins are inhibited by immunity proteins has been provided by atomic structures, with associated biochemical studies, of various effector : immunity complexes (Table 1, Fig. 2). Together, these studies show that the immunity protein normally binds to the toxin to block the active site, with the exception of Tle4/Tli4 where Tli4 binding away from the active site prevents an activating conformational change in Tle4 (Lu *et al.*, 2014b). The structures of the immunity proteins and the stoichiometry of the effector : immunity complexes vary considerably, with many of the immunity proteins displaying previously undescribed protein folds. Work in *S. marcescens* further revealed that two related Tae4 family effectors, Ssp1 and Ssp2, are neutralized by structurally unrelated immunity proteins, Rap1a (Tai4a^SM^) and Rap2a (Tai4^SM^) (Srikannathasan *et al.*, 2013). These two effector : immunity pairs are encoded together with two other immunity proteins of the Tai4 family, representing examples of ‘orphan’ immunity proteins, which do not confer protection against endogenous effectors, but rather may provide protection against exogenous effectors from other organisms (English *et al.*, 2012, Srikannathasan *et al.*, 2013).

## Evolutionary considerations

Several interesting comparisons can be made between T6SS‐delivered anti‐bacterial toxins and other systems that mediate bacterial antagonism. Specialized effectors can be considered as examples of polymorphic toxins, where the N‐terminal domain of the protein determines secretion or localisation (for T6SS, a core VgrG or PAAR domain) and the final C‐terminal domain is a variable toxin domain. Furthermore, like other polymorphic toxins, specialized effectors are associated with immunity proteins specific to their toxin domains and encoded immediately downstream (Zhang *et al.*, 2012). More broadly, many toxin domains, particularly nuclease and pore‐forming domains, appear to be shared between different antagonistic systems, including polymorphic contact dependent toxins (such as Cdi and T6SS toxins) and diffusible bacteriocins. Thus, certain T6SS‐associated effectors may have evolved from, or been a source of, toxins associated with other systems. Another common feature of polymorphic toxins is the presence of genes encoding ‘orphan’ C‐terminal toxin domains and immunity proteins downstream of the full length toxin‐immunity locus (Poole *et al.*, 2011; Zhang *et al.*, 2012). These are believed to be remnants of past homologous recombination events, when new toxin domains and immunity proteins have been horizontally acquired and exchanged into the polymorphic toxin locus. These events would allow facile acquisition and immediate deployment of new toxins, as has been demonstrated for Rhs in *Salmonella typhimurium.* Specifically, extended passage yielded an evolved subpopulation able to inhibit parental cells by deployment of an orphan Rhs C‐terminal domain, following the fusion of this domain with the main ancestral Rhs protein through a recombination event (Koskiniemi *et al.*, 2014).

Horizontal acquisition is also likely to be a feature of cargo effector‐immunity pairs. Considerable variation in effector‐immunity complement is observed between different strains, and closely related effectors are frequently found in distantly related organisms (Barret *et al.*, 2011; Russell *et al.*, 2012; Russell *et al.*, 2013; Unterweger *et al.*, 2014). Indeed, consistent with facile movement of effector‐immunity loci between strains and organisms, the T6SS appears to be very effective in intra‐species as well as inter‐species competition (Unterweger *et al.*, 2014; Alcoforado Diniz and Coulthurst, 2015). Recent work has further suggested that specific, genetically linked accessory proteins may co‐evolve with effectors, containing conserved regions to mediate interaction with T6SS proteins and variable regions to interact with the cognate effector (Unterweger *et al*., 2015). Additionally, a study of DNA transfer between *Bacteroidales* species demonstrated for the first time the transfer of a putative T6SS locus between strains within a natural human ecosystem (Coyne *et al.*, 2014).

An intriguing question is which came first, anti‐bacterial or anti‐eukaryotic T6SSs? Perhaps the identification of several effectors, including phospholipases, which are active against both kingdoms, may help to explain the expansion from one to the other. Was ‘cargo’ or ‘specialized’ the original mode of effector delivery? Certainly, the frequent location of cargo genes immediately 3’ of their cognate VgrG gene is suggestive that they once were, or in the future could be, fused into a single specialized protein. But even highly efficient anti‐bacterial cargo effectors are not the end of the evolutionary line. Recently, it has been shown that genes encoding T6SS anti‐bacterial effectors can be further transferred to eukaryotic organisms to augment their innate immune system (Chou *et al.*, 2015). Overall, it is tempting to speculate that acquisition of new T6SS effector‐immunity pairs may be a common way in which new isolates of increased competitive fitness against closely or distantly related bacteria arise. Similarly, acquisition of new anti‐eukaryotic effectors may extend the range or efficiency of host interaction, although whether the target specificity of effectors is the sole factor in determining the type of cells targeted by a particular T6SS remains to be determined.

## Conclusions

The T6SS machinery delivers a diverse range of effector proteins into both prokaryotic and eukaryotic cells. These toxic effectors are frequently used to kill or incapacitate surrounding bacteria in order to thrive in the polymicrobial environment, whilst possession of cognate immunity proteins prevents self‐intoxication. Alternatively, the T6SS can be a major direct virulence determinant, delivering effectors that interfere with biological activities or integrity of eukaryotic host cells during infection. It is likely that acquisition of T6SS effectors can play an important role in the continual evolution of bacterial competitiveness, even transferring to eukaryotic lineages on occasion. Looking ahead, there must surely be a great number of T6SS‐delivered effectors still to be discovered, with the promise of new biology to be explored. Other future areas of research include questions such as the following: If one bacterium secretes multiple T6SS effectors, are they all delivered simultaneously and do they act co‐ordinately in recipient cells? How do effectors reach different compartments in target cells? What is the role of anti‐bacterial T6SSs in the healthy and diseased microbiome and what non‐antagonistic roles might their effectors have? Long‐term, we anticipate that understanding how T6SS effector proteins are delivered and act in the target cell may aid the identification of novel targets for the development of new anti‐bacterial therapies.
